# Evaluating the use of stable isotope analysis to infer the feeding ecology of a growing US gray seal (*Halichoerus grypus*) population

**DOI:** 10.1371/journal.pone.0192241

**Published:** 2018-02-21

**Authors:** Jacob E. Lerner, Kathryn Ono, Keith M. Hernandez, Jonathan A. Runstadler, Wendy B. Puryear, Michael J. Polito

**Affiliations:** 1 University of New England, Department of Marine Sciences, Biddeford, Maine, United States of America; 2 Lousiana State University, Department of Oceanography and Coastal Sciences, Baton Rouge, Louisiana, United States of America; 3 Tufts University, Cummings School of Veterinary Medicine, Boston, Massachusetts, United States of America; Universidade de Aveiro, PORTUGAL

## Abstract

Gray seals *(Halichoerus grypus*) have been rapidly recolonizing the Northeast US coast, eliciting concern from the fishing industry. However, the ecological effect of this recovery is still unknown and as such, research is needed to better understand how the diet composition of gray seals in US waters will contribute to the ecological impact. While previous research on seal diets has focused on the analysis of hard prey remains, stable isotope analysis presents an alternative method that can be used to describe marine mammal diets when direct observation is impossible. To address this issue, we used stable isotope analysis of gray seal pup vibrissae and lanugo from Monomoy Island, Cape Cod, MA during the 2015/2016 winter breeding season to estimate adult female diet composition during pregnancy. Stable isotope mixing models (SIMM) suggested adult female gray seals were consuming greater amounts of cephalopod prey and less sand lance than previously indicated from analysis of hard prey remains. However, using SIMMs to estimate the diet composition of gray seals remains difficult due to the large number of isotopically similar prey species and uncertainty in tissue-specific, stable isotope trophic enrichment factors. Even so, by combining prey sources into ecologically informative groups and integrating prior information into SIMMs it is possible to obtain additional insights into the diet of this generalist predator.

## Introduction

The gray seal’s (*Halichoerus grypus*) return from near extirpation to the Northeastern United States has been celebrated by conservationists but has reignited controversy with some in the fishing industry [1–5]. This is because there is the potential for these seals to be predators of many fish species in economically significant fisheries [6,7]. Further, their recolonization comes at a critical time in the ecosystem, as the northwest Atlantic is experiencing rapid warming [8]. The gray seals in the US differ in distribution and foraging region from the nearby Canadian population on Sable Island, Nova Scotia [5,9,10]. As such, determining the diet composition of gray seals in US waters is critical to understanding the economic and ecological impact of their recovery and preparing management strategies for the future [6,7,11].

Previous diet studies of gray seals often focused on the analysis of scats and stomach contents [1,9,12]. However, both of these approaches have known limitations [9,13,14]. Scats can only be collected at haul-out sites or from breeding colonies [9]. If these haul-out sites are only located near shore, as they are in the case of the US population, this can bias the analysis towards near shore prey species [12,15,16]. Furthermore, heavily digested material cannot be identified in some scat samples and stomach contents [9,12,14,17–19]. Scat analysis may also underrepresent soft-bodied prey and feeding that avoids major components of hard part analysis [17,19,20]. Stomach content analysis may only include animals participating in offshore feeding captured in the by-catch (i.e. unintentionally netted by commercial fisheries), and is typically skewed towards juvenile individuals [9,21].

Stable isotope analysis represents an alternate approach to quantify marine predator foraging ecology and diets [22,23]. This is because the stable carbon and nitrogen isotope composition in tissues act as a record of an animal’s diet during tissue synthesis [24,25]. With these two elements, stable isotope analysis utilizes the ratio of heavy to light naturally occurring stable isotopes (SI) of carbon (^13^C/^12^C) and nitrogen (^15^N/^14^N) within an animal’s tissues, and these standardized ratios are expressed as δ^13^C and δ^15^N respectively [22,25,26]. During tissue synthesis, the heavier isotopes of carbon and nitrogen are preferentially assimilated into a consumer’s tissues in a process called discrimination [24,25,27]. The amount by which δ^13^C and δ^15^N values increase in a consumer with each trophic transfer is referred to as a trophic enrichment factor (TEF) and can vary depending on consumer species, prey item and consumer tissues [16,28]. δ^13^C values can provide insight into the use of basal carbon sources in an animal’s food web and δ^15^N values provide a proxy for its trophic level [22,24,29,30]. Stable isotope mixing models (SIMM) integrate the stable isotope values of consumers and their prey to quantify the proportion of each prey species in a consumer’s diet [16,31]. However, for these models to be successful, adequate isotopic spacing of prey and accurate tissue-specific isotopic trophic enrichment factors are necessary [32,33].

While past studies have used gray seal SI values to draw conclusions about sexual and ontogenic foraging behaviors, none have attempted to use a SIMM to reconstruct their diet composition [25,29,34]. In addition, to our knowledge no studies have used SI values to examine the foraging ecology of gray seals in US waters [34]. As such, the objective of our study is to evaluate the ability of stable isotopes analysis and SIMMs to provide high confidence estimates of the diet composition of gray seals in US waters. We focus our isotopic analyses on pup tissues developed *in-utero*, vibrissae and fur (lanugo), thus serving as a potential proxy for the mother’s diet [35]. While this approach has not been used before in gray seals, pup tissues have been used to examine adult female diets in other phocid and otariid seal species using SIMMs [16,22,36]. Specifically, the primary aims of this study were to: (1) examine the interchangeability of pup vibrissae and lanugo as proxies of maternal diet, (2) quantify the SI values of potential gray seal prey species, (3) test the applicability, to gray seals, of commonly used TEFs, and (4) use SIMMs to estimate the diet composition of gestating adult female gray seals in US waters.

## Methods

### Sample collection

Gray seal samples were collected under permit 17670 issued to the National Oceanographic and Atmospheric Administration (NOAA), National Marine Fisheries Service Northeast Science Center. We collected vibrissae from 25 weaned pups from Monomoy Island (41.5500° N, 70.0000° W) during January 2016. A single vibrissae was clipped from the base of the muzzle on the right side of each sampled pup. Of those 25 pups, 19 also had lanugo samples collected. Prey samples were acquired through the 2016 NOAA spring bottom trawl off the coast of Massachusetts.[5,9]. Pups were randomly sampled from stage III and stage IV pups (i.e. weaned pups that had yet to fully shed their lanugo) present on the island. Two to eight individuals each of the ten most important prey species were acquired based on previous scat/stomach diet analyses of Northwest Atlantic gray seal populations [9,10,12] (Table 1). Specifically, these ten prey species represented more than 90% of dietary biomass in analyses of gray seal scat and stomach samples in New England [9]. As gray seal scat data is likely indicative of inshore foraging and stomach contents of offshore foraging, the selected species represent the dominate prey available to seals in this region, and it is unlikely a major prey item is missing from our analysis [9].

**Table 1:** Prey species SI values.

Species	*n*	δ^13^C (‰)	δ^15^N (‰)	C/N
Squids				
Longfin squid, *Doryteuthis pealeii*	5	-17.2±0.3^bc^	10.1±1.2^bc^	3.1±0.0
Northern Shortfin Squid, *Illex illecebrosus*	5	-17.1±0.4^bd^	8.0±0.5^a^	3.1±0.0
Sand Lance				
Sand Lance, *Ammodytes* spp.	7	-19.9±0.5^a^	9.5±1.7^ab^	3.1±0.0
Demersal Fishes				
Atlantic Cod, *Gadus morhua*	5	-17.6±0.7^b^	12.8±0.4^d^	3.1±0.0
Red Hake, *Urophycis chuss*	5	-15.5±0.4^e^	15.4±0.4^ef^	3.1±0.0
Silver Hake, *Merluccius bilinearis*	5	-15.8±0.5^de^	16.0±0.3^f^	3.0±0.0
Thorny Skate, *Amblyraja radiata*	2	-15.8±0.7^cde^	12.0±0.8^cd^	3.2±0.1
White Hake, *Urophycis tenuis*	5	-17.0±1.2^bd^	12.9±0.3^d^	3.1±0.0
Winter Flounder, *Psuedopleuronectes americanus*	5	-16.5±0.6^be^	14.9±0.6^ef^	3.1±0.0
Winter Skate, *Leucoraja ocellata*	5	-16.9±0.4^bd^	13.9±0.2^de^	3.0±0.0

Mean±SD δ^13^C and δ^15^N stable isotope values for potential gray seal prey species collected from the Northwest Atlantic, May-June 2016. Prey species that share a superscript within a column are not significantly different at the p = 0.05 level.

### Sample preparation

We used pup vibrissae and lanugo tissues developed *in-utero* as proxies of maternal diet. Vibrissae begin growing in gray seal fetuses between 2–3 months of active gestation and continue to develop over the next 6–7 months until birth [35]. For pups born in December—January, vibrissae therefore reflect summer/fall foraging seasons. Pups are born with vibrissae roughly 2–3 cm long and grow at a rate of .0025cm/day to about 5-7cm during the lactation period [35–37]. The maximum growth rate recorded in phocid pups was 0.087 cm/day in the bearded seal (*Erignathus barbatus*) [36]. Therefore, for a 4–6 week old weaned pup with 5–7 cm long vibrissae, a maximum of 3.6 cm of new vibrissae growth could have occurred since birth, leaving the distal 2–3 cm to reflect *in utero* growth [16,36]. Thus for all vibrissae samples, we saved a distal 2 cm section for analysis. In gray seals, lanugo begins growing around 3–4 months of gestation and grows up until birth [35,38,39]. Like vibrissae, lanugo reflects a summer/fall foraging season. Prior to isotopic analysis, we rinsed vibrissae and lanugo samples in a 2:1 chloroform:methanol solution for 24 h and air dried for a further 24 h.

We freeze-dried, and pulverized roughly 1.5g samples of muscle tissue from each of the potential prey species. Individuals for all species were sampled two times except elasmobranch species, which were sampled four times. Following the protocol of Kim and Koch [40] we performed a lipid extraction (LE) using a 2:1 chloroform:methanol on one of the samples. Both lipid extracted and non-lipid extracted (NLE) muscle tissue for all prey species were processed for SI analysis. Elasmobranch species underwent an HCl and non-HCl treatment following the protocol of Kim and Koch [40] to remove cartilaginous tissue from the muscle that could interfere with the δ^13^C signature. For most prey species LE treatments were used for δ^13^C analysis and NLE treatments for δ^15^N analysis, except in elasmobranch muscle where HCl and LE treated muscle was used for δ^13^C analysis.

### Stable isotope analysis

We flash-combusted (Costech ECS4010 elemental analyzer) 2 cm vibrissae sections (0.3–1.6 mg) and approximately 0.8 mg of lanugo and homogenized prey muscle tissue samples loaded into tin cups and analyzed for carbon and nitrogen isotopes (δ^13^C and δ^15^N) through interfaced Thermo Finnigan Delta Plus XL continuous-flow stable isotope ratio mass spectrometer. Raw δ values were normalized on a two-point scale using glutamic acid reference materials with low and high values (i.e. USGS-40 and USGS-41). Sample precision based on repeated reference material was 0.2‰ and 0.1‰ for δ^13^C, and δ^15^N, respectively.

Stable isotope ratios are expressed in δ notation in per mil units (‰), according to the following equation: δX = [(R_sample_ / R_standard_) - 1] ∙ 1000. Where X is ^13^C or ^15^N and R is the corresponding ratio ^13^C /^12^C or ^15^N /^14^N. The R_standard_ values were based on the Vienna PeeDee Belemnite for δ^13^C and atmospheric N_2_ for δ^15^N.

### Statistical analysis

We used Pearson’s correlation tests to examine the relationship between vibrissae and lanugo SI values and paired t-tests to test for differences in SI values between tissues. Unpaired t-tests were used to test for differences in tissue SI values between sexes. An ANOVA with Tukey’s HSD post-hoc comparisons were used to examine differences in the SI values of prey species. All statistical analyses were performed in R (Version 3.3.1).

### SIMM analysis

Mixing models cannot be fit blind; background knowledge of consumer diet is necessary to choose appropriate food sources to fit into the model [32,41]. Even with this knowledge, models where the number of contributing sources is greater than or equal to one plus the number of isotopes used are fundamentally undetermined with multiple existing solutions [32,41]. As such, Bayesian SIMMs have been developed to estimate the probability distributions of multiple prey source contributions to a consumer while accounting for the observed variability in prey and consumer isotopic values, TEFs, and elemental concentration [42]. Even so, the discriminatory power of SIMMs generally decreases with the number of prey sources, is strongly influenced by the isotopic separation of prey sources, and can be biased by missing prey sources [32,43]. When the above issues lead to reduced discriminatory power, dietary data from independent sources can be incorporated as informative priors to improve the accuracy and precision of SIMMs [44].

Given the issues raised above we used *a priori* source-partitioning methods to combine prey species into statistically and ecologically relevant prey groups prior to their incorporation into SIMMs based on their SI values. First, similar to Hindell et al [36], a hierarchical clustering analysis utilizing Ward’s method, accounting for variation in both δ^13^C and δ^15^N, was applied to cluster the ten prey species into six prey groups as the discriminatory power of SIMM can decline markedly above six or seven sources [32]. Second, following Edwards et al [45] and Philips et al [41], we further reduced these six prey clusters into three distinct groups based on ecological similarities in habitat and functional group to determine if condensing prey groups increased the discriminatory power of SIMMs predictions, albeit at a lower taxonomic resolution.

Stable isotope mixing models can be highly sensitive to the TEFs employed [46]. As species and tissue-specific TEFs are not currently available for fetal gray seals, δ^13^C and δ^15^N TEFs were chosen from three potential sources: averaged ecosystem TEFs developed from Post [47] derived from a meta-analysis of laboratory and field observations of trophic fractionation across a wide range of ecosystems, a mean of all experimentally determined TEFs for phocid seals [48,26,27], and meta-analysis derived TEFs from determined using the package Stable Isotope Discrimination Estimation in R (SIDER) [49] (Table 2). The SIDER tool uses a Bayesian linear modeling approach to predict a species TEF value based on their physiology, phylogenetic relationships and experimental variation from published literature [49]. Following the method of Smith et al [50] a Monte Carlo simulation of stable isotope mixing polygons was used to select the most appropriate TEFs [16,42]. Consumer vibrissae and lanugo isotope values were independently fit to mixing model polygons created using prey cluster δ^13^C and δ^15^N values adjusted for each TEF, incorporating both TEF and prey SI value uncertainty, over 1500 iterations. TEFs were chosen for incorporation into SIMMs by selecting the values that provided the best fit (i.e. fewest number of consumer signatures near the edge of the polygon’s 95% confidence interval).

**Table 2:** TEFs values evaluated for use in this study.

TEF Value Source	Vibrissae		Hair	
	δ^15^N	δ^13^C	δ^15^N	δ^13^C
Experimentally Derived				
*Hobson et al* [26]^*a*^	2.8±0.1	3.2±0.2	3.0±0.4	2.8±0.5
*Lesage et al* [27]^*b*^	NA	NA	2.3±.0.8	2.3±0.1
*Beltran et al* [48]^*c*^	3.2±0.5	3.4±0.5	NA	NA
*Average*	3.0±0.4	3.3±0.4	2.9±0.5	2.7±0.5
Post [47]*	3.4±0.98	0.4±1.3	3.4±0.98	0.4±1.3
SIDER [49]	2.6±1.2	2.4±1.3	2.6±1.2	2.4±1.3

TEF values calculated as a mean±SD of all experimentally determined TEFs for phocids (adult vibrissae and hair), the standard values determined by Post [47] and those determined by the SIDER program.

* denotes TEF value based of mixing model polygon results.

^a^ *n* = 7 for vibrissae (harbor, harp and ringed seal), *n* = 10 for hair (harbor, harp and ringed sea)

^b^ *n* = 2 for hair (gray seal)

^c^ *n* = 8 for vibrissae (monk, elephant, harbor, spotted and ringed seal)

Stable isotope mixing models were run using the MixSIAR package [51]. Separate models were run for each tissue (vibrissae and lanugo) and for each prey cluster grouping (six prey sources and three prey sources). Additional variants of the three prey sources models were run using diet composition data from Ampela’s [9] analysis of gray seal scats in New England as informative priors as opposed to equally weighted priors to assess the ability of prior information to increase the resolution of SIMM outputs. Priors were adjusted for normalized dietary contributions of 1.4% for squid species, 53.3% for sand lance, and 40.6% for a combination of all remaining ‘demersal fishes’ in the analysis [9]. Each model ran over three Markov Chain Monte Carlo chains of 1,000,000 iterations, thinned by 500, and with an initial discard of the first 500,000 iterations. Bayesian model outputs are reported as median and 95% credible intervals.

## Results

### Gray seal stable isotope values

Between vibrissae and lanugo from the same individual, there was significant and positive correlation in δ^13^C (r = 0.91, p<0.05) and δ^15^N (r = 0.86, p<0.05) values. Despite this, both δ^15^N and δ^13^C values significantly differed between vibrissae and lanugo (δ^15^N: t = 2.88 p<0.05; δ^13^C: t = -3.16, p<0.05; Table 3). Gray seal tissue δ^15^N (vibrissae δ^15^N: t = -0.57, p = 0.57; lanugo δ^15^N: t = 0.06, p = 0.95) and δ^13^C values (vibrissae δ^13^C t = -0.28, p = 0.79; lanugo δ^13^C: t = -0.38, p = 0.71) did not differ between sexes.

**Table 3:** Vibrissae and lanugo SI values.

Tissue	Sex	*n*	C/N	δ^13^C (‰)	δ^15^N (‰)
Vibrissae	Male	13	3.0±0.1	-16.3±1.6	15.4±0.9
	Female	11	3.0±0.1	-16.4±1.4	15.2±0.7
	All	25^1^	3.0±0.1	-16.4±1.5	15.3±0.8
Lanugo	Male	9	3.0±0.1	-16.4±1.6	15.6±1.2
	Female	9	3.0±0.1	-16.6±1.5	15.6±0.9
	All	19^1^	3.0±0.1	-16.6±1.5	15.6±1.0

Mean±SD C/N ratio, δ^15^N, and δ^13^C values of vibrissae and lanugo collected from gray seal pups on Monomoy Island, January 2016.

^1^One sample with sex unknown

### Prey stable isotope values

Muscle tissue δ^13^C and δ^15^N values differed among the ten prey species (δ^15^N: F (9, 39) = 50.32, p<0.05; δ^13^C: F (9, 39) = 25.13, p<0.05). Northern shortfin squid (*Illex illecebrosus*) and longfin squid (*Doryteuthis pealeii*), had δ^13^C values near the middle of the prey distribution but had the first and third lowest δ^15^N values respectively, isolating them from other prey (Table 1). Sand lance (*Ammodytes* spp.) had the lowest δ^13^C values and second lowest δ^15^N values distinguishing it from other prey (Table 1). At the opposite end of the spectrum, red hake (*Urophycis chuss)* and silver hake (*Merluccius bilinearis*) both had higher δ^13^C and δ^15^N values than all other species and were not significantly different from one another (p>0.05). Apart from these three distinct groups, the middle of the distribution contained both winter skate (*Leucoraja ocellata*), with high δ^15^N and low δ^13^C values, and thorny skate (*Amblyraja radiata*) with conversely high δ^13^C and a low δ^15^N values (Table 1). Also within this group were white hake (*Urophycis tenuis)*, Atlantic cod (*Gadus morhua*) and winter flounder (*Psuedopleuronectes americanus*), which had δ^13^C and δ^15^N values near the upper-middle end of the distribution and, except for winter flounder δ^15^N values, were not significantly different from one another (p>0.05).

### Cluster analyses and TEF choice

Our hierarchical cluster analysis assigned the ten prey species into six groupings based on their isotopic values: 1) sand lance, 2) northern longfin squid and shortfin squid, 3) red hake and silver hake, 4) winter flounder and winter skate, 5) thorny skate, and 6) Atlantic cod and white hake. Our second grouping method assigned prey species into three groups: 1) sand lance, 2) northern longfin squid and shortfin squid, and 3) a ‘demersal fishes’ group that included red hake, silver hake, winter flounder, winter skate, thorny skate, Atlantic cod, and white hake.

**Fig 1:**
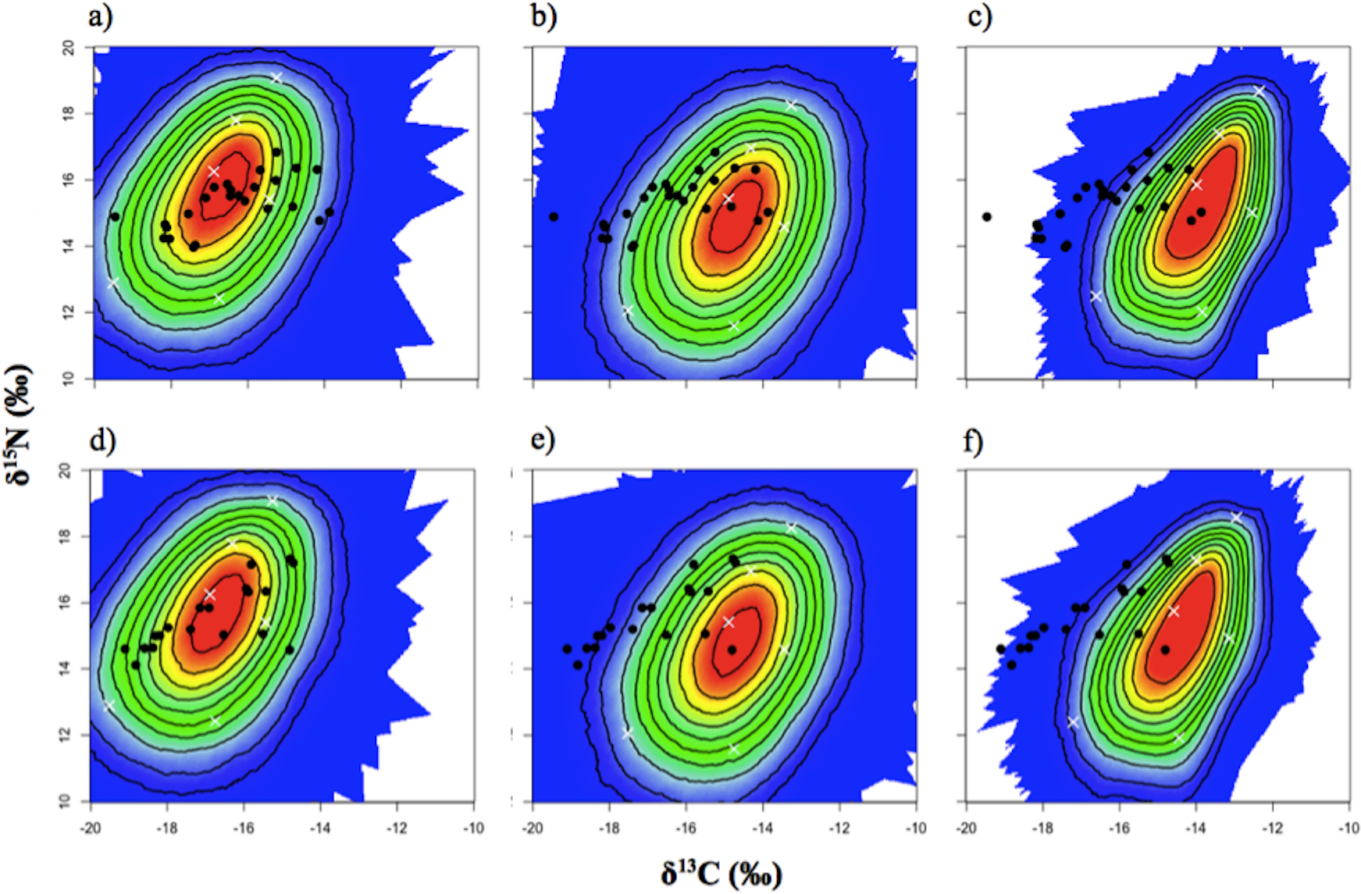
Mixing model polygon results. Stable isotope mixing model polygons for gray seal vibrissae (a,b,c) and lanugo (d,e,f) relative to six potential prey species groups. Black dots: consumer SI signatures. White crosses: average source SI signatures adjusted for TEF values. Colored region represents the 95% confidence interval. Probability contours are at the 5% level. TEF values derived from a,d) Post[47], b,e) SIDER; c,f) Experimentally derived [48,26,27].

TEF values from Post [47] were chosen as the most appropriate following a quantitative assessment of multiple potential TEF values for both vibrissae and lanugo using stable isotope mixing model polygons (Fig 1). For both tissues, the TEF values from Post [47] had the greatest number of consumer points within the confidence intervals of the mixing polygon created by prey source groups.

### SIMM analyses

In general there was broad overlap in the predicted mean diet composition and 95% credibility intervals in all model variants regardless of the consumer tissue of interest (vibrissae vs. lanugo; Table 4). Models that included six prey clusters were poorly resolved with 95% credibility intervals overlapping substantially across all prey sources. Six prey source SIMMs predicted mean diet contribution ranging from 11.5 to 23.5% with a slightly more uniform distribution across species predicted by lanugo relative to vibrissae (Table 4).

**Table 4:** SIMM results.

Model structure, prey sources	Mean diet composition (95% credibility intervals)	
	Vibrissae	Lanugo
*Six source models*		
Squid	23.5 (5.7–41.2)	16.6 (2.2–33.6)
Sand Lance	11.5 (1.3–25.3)	17.3 (3.4–32.6)
Atlantic Cod and White Hake	15.8 (1.3–37.5)	18.8 (1.6–44.6)
Red Hake and Silver Hake	13.1 (1.3–29.0)	14.3 (1.3–31.7)
Thorny Skate	21.9 (2.5–46.8)	16.1 (1.4–38.9)
Winter Flounder and Winter Skate	14.2 (1.2–33.0)	16.8 (1.5–38.5)
*Three source models*		
Squid	35.5 (17.7–48.3)	23.9 (5.7–41.8)
Sand Lance	9.7 (0.4–23.7)	18.8 (3.4–35.6)
Demersal Fishes	54.8 (43.9–63.6)	57.3 (46.3–67.8)
*Three source models with priors*		
Squid	22.7 (0.0–42.5)	4.0 (0.0–23.8)
Sand Lance	20.6 (4.0–42.2)	35.0 (17.9–47.5)
Demersal Fishes	56.7 (46.9–66.7)	61.0 (49.7–71.7)

Percent diet composition with 95% credibility interval in parenthesis for prey groupings as estimated by the SIMMs based on vibrissae and lanugo tissue sampled from gray seal pups on Monomoy Island, January 2016.

Better resolution (i.e precision) was provided by SIMMs that only included three prey clusters. Three prey source SIMMs predicted that approximately half of seal’s diets were comprised of other ‘demersal fish' relative to squid and sand lance, with little to no overlap in 95% credibility intervals (Table 4). While the relative predicted mean contribution of squid vs. sand lance was consistently higher in three source models using vibrissae, 95% credibility intervals overlapped between these two prey sources in all models. Using scat data as a Bayesian prior to inform the three source models consistently resulted in reductions in the predicted mean contribution of squid vs. sand lance, though 95% credibility intervals still overlapped in these two prey sources (Table 4).

## Discussion

### Gray seal and prey stable isotope values

We found no differences in tissue stable isotope values between male and female pups. Evidence from the eastern Atlantic suggests that male pups are born larger than female pups and are more energetically costly to gestating females [52,53]. However, studies from a more closely related gray seal population on Sable Island have failed to find similar results of greater male size at birth [54,55]. As such, our results may support the notion of equal male/female fetal investment in gray seals. Even so, given the level of isotopic overlap in possible prey species, we cannot completely rule out the possibility that diet composition might vary to some degree depending on the sex of the fetus.

**Fig 2:**
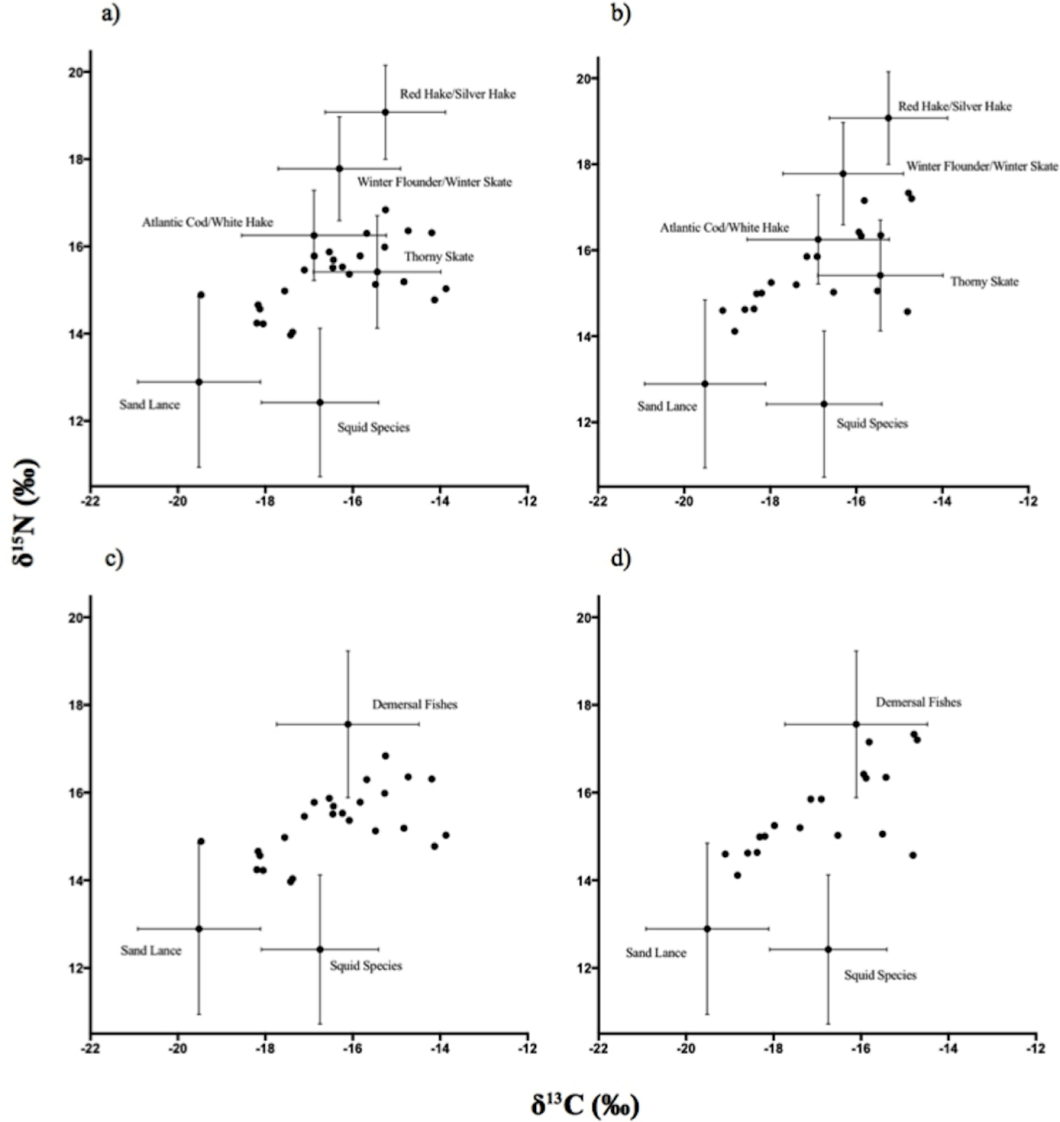
δ^15^N and δ^13^C bi-plots of gray seal and prey SI values adjusted for Post [47] TEFs. δ^13^C and δ^15^N stable isotope values of gray seal pup vibrissae (a,c) and lanugo (b,d) tissues relative six (a,b) and three (c,d) prey source groupings. Prey source are presented as mean±SD and adjusted for Post [47] TEFs.

The positive correlation found between tissues suggests that vibrissae and lanugo can serve interchangeably as proxies of maternal dietary isotopic signatures during gestation. However, significant differences between tissues for both δ^15^N and δ^13^C values indicate that they may be capturing different time frames of maternal foraging. Lanugo δ^13^C values were 0.41‰ (±3.0) lower than vibrissae, and lanugo δ^15^N were 0.36‰ (±1.8) higher than vibrissae. These two keratinous tissues presumably share similar TEFs due to their chemical similarity [39,48,26,27]. Lanugo is only grown *in-utero* and therefore should maintain a maternal signature [56]. Both tissues develop slowly throughout gestation and have major overlapping periods of growth yet vibrissae begin development 1–2 months prior to lanugo [35,39]. The discrepancies in SI values may reflect these different time frames of growth. In addition, lanugo values were more centrally located within the mixing model polygons in bivariate isotopic space (Fig 2). This made the lanugo model more sensitive to prior information (i.e posterior distribution more closely reflected prior distribution) than the vibrissae model (Table 4). Aside from this example, lack of congruity between the tissues was small enough not to cause major differences between the SIMMs (Table 4). Thus, lanugo may be preferred over vibrissae when trying to reduce invasiveness and maximize sample mass due to its ease of sampling.

The stable isotope values of potential prey species sourced from the Northwest Atlantic illustrated substantial isotopic overlap between species. Among demersal ground fish, we found no statistical isotopic difference between Atlantic cod and white hake and no statistical difference between red hake and silver hake suggesting these species share similar trophic niches. Northern shortfin squid and longfin squid, though unique from many other species were also statistically isotopically identical to one another for δ^13^C but not for δ^15^N. No species were isotopically unique in bivariate isotopic space, though sand lance, with only two significant relationships, had the fewest statistical overlaps. These results indicate a general lack of spacing between many of the potential gray seal prey species in the Northwest Atlantic, and in some cases large variation within individual prey species. While difficult to assess, it is possible that the small sample sizes analyzed for some prey groups impacted the ability to provide precise estimates of prey SI values and contributed to higher uncertainty of the diet analysis. However, our findings generally agree with the results of an analysis of stable isotope values of common species from Georges Bank, Northwest Atlantic, albeit with slight differences in δ^15^N and δ^13^C values for white hake and silver hake and a larger sample size for species analyzed in our study [57].

### Combining prey sources

Our research illustrates one of the major challenges in using SIMMs to infer the diet composition of gray seals: They are a generalist predator feeding on several isotopically similar prey sources. Similar to studies of other generalist consumers, the large number of possible prey species and the degree of overlap between these species it was necessary to combine sources prior to including them in SIMMs [16,36,45]. However, while combining prey sources can increase the predictive strength of SIMMs, the loss of taxonomic resolution can limit the model’s interpretation and applicability [41].

Our study used cluster analyses to isolate the most statistically similar of the ten likely prey species into six groups and further reduced that into three possible prey clusters. Models that included six prey clusters provide little interpretative value due to the large overlap in 95% credibility intervals. However, combining sources into three prey clusters allowed us to draw more robust conclusions, though at lesser taxonomic resolution. Specifically, three source models provide better constrained estimates into the consumption of the ‘demersal fishes’ group relative to the other possible prey sources. This suggests to us that for the gray seal, with its plentiful dietary inputs, combining sources into ecologically informative groups may be the best way to derive useful information from SIMMs. For example, the approach used here provides insights into seal’s foraging into non-commercial species such as sand lance and squids.

### Incorporating informative priors

One unique feature of Bayesian SIMMs is the ability to incorporate information from previous dietary knowledge as informative priors which can add additional refinement to model outputs [32,51,58,59]. Previous dietary knowledge incorporated into SIMMs may come from, but is not limited to, sources such as stomach content, scat, or prey DNA [44,60,61]. However, while integrating complementary dietary techniques in SIMMs can provide better estimates of the actual diet of consumers [44], there are potential trade-offs. For example, Franco-Trecu et al [60] found that incorporating scat analysis data into SIMMs used to estimate the diet compositions of the South American fur seals (*Arctocephalus australis*) and sea lions (*Otaria flavescens*) improved the precision in the estimated diet composition at the risk of inducing biases inherent to the original scat analysis in the estimates.

Incorporating data from scat analysis into our gray seal SIMMs consistently decreased the importance of squid relative to sand lance in comparisons to uninformed models but surprisingly did not improve the relative precision of these estimates (Table 4). This may have been driven by the large amount of isotopic variation incorporated into the prey groupings [44]. However, it is important to note that our source of prior information (e.g. scat analysis) may be biased towards inshore prey, such as sand lance, relative to other prey species [9,12]. Previous studies have shown that gray seal scat analysis in New England contains a dietary record of only 24–48 hours of foraging, occurring within 80km of sampled haul-out sites, and is likely reflective of inshore feeding [9]. As such, similar to Franco-Trecu et al [60] we caution that to draw reliable results from SIMMs using informative priors, there must be a high confidence in the accuracy of the complementary source data used to avoid inducing additional biases into model results.

### Selecting TEF values

Another challenge when using SIMMs to infer the diet composition of gray seals is selecting appropriate TEFs. In our study, TEF selection was determined by the ‘best-fit’ mixing model polygon following the procedure outlined by Smith et al [50]. As the prey species used in this analysis reflect the dominate prey species consumed by gray seals in New England (i.e. greater than 90–95% of dietary biomass) [9] it is unlikely there were major unrepresented prey species in our analysis. Regardless, the high degree of variability in some prey taxa likely led to the large 95% confidence interval observed for our mixing model polygons (Fig 1). Surprisingly, the averaged ecosystem TEFs of Post [47] were the best fit and outperformed TEFs determined experimentally through a combination of captive feeding trials on phocid seals and those produced via the SIDER tool [48,26,27,49] (Fig 1).

This suggest the generic TEFs of Post [47] may be the most accurately calibrated to the isotopic modification between gray seal pup vibrissae and lanugo tissues relative to their mother’s diet. There are several possible reasons for this result. First, it is possible that captive experimental conditions under which phocid TEFs have been determined in previous studies [48,26,27] are not representative of the environmental conditions that occur in the wild. This can occur either due to less varied diets and subsequent isotopic routing or lower energetic demands of animals in captivity [44,62]. Also, averaged TEFs from Post [47] may have been a better fit because the exchange of isotopes between maternal gray seals and pup tissues *in-utero* may not be identical to the same relationship between the mother and her own tissues [62]. Stricker et al [62] found that pup vibrissae grown *in-utero* were 0.8‰ higher for δ^15^N and 0.4‰ lower for δ^13^C than paired maternal vibrissae in the otariid Steller sea lion, *Eumetopias jubatus*. These results were similar to a paired mother/pup vibrissae study by Lowther and Goldsworthy [63] on Australian sea lions, *Neophoca cinera*, which found differences between *in-utero* and maternal vibrissae of 1.2‰ higher for δ^15^N and 0.2‰ lower for δ^13^C [63]. Accordingly, the Post [47] TEFs had higher δ^15^N and lower δ^13^C values relative to experimentally and SIDER derived TEFs (Table 2). Thus this result may be expected if *in-utero* isotopic fractionation of tissues in gray seals was similar to Australian sea lions (i.e higher δ^15^N and lower δ^13^C values relative to adult TEFs). Also noteworthy, the Post [47] TEF values were in close agreement with the averaged TEF values chosen by Hindell et al [36] in their use of *in-utero* pup vibrissae on another phocid, the bearded seal, suggesting their may be phylogenetic similarity in *in-utero* TEFS. Similar to Post [47], the TEFs from Hindell et al [36] are derived from a standard environmental system value and not from experimental data on in-utero tissue development in phocid pups [64].

### Gray seal diet predictions

While both six and three prey source models results often had substantial overlap in 95% credibility intervals between prey groups, some inferences in the diets of gestating adult female gray seals are still possible. For example, all SIMM predictions suggest that female gray seals have a larger proportion of cephalopod prey in their diets and a lower reliance on sand lance than had been previously reported for gray seal populations [9,12,65] (Fig 2; Table 4). In addition, these results also support the notion that US gray seals are generalist consumers, foraging on a mixture of commercial and non-commercial fish stocks. For example, SIMMs with six prey sources suggested that between 30–34% of diet had the potential to be derived from commercially valuable species such as Atlantic cod and winter flounder, though there was substantial uncertainty around these predictions. When considering the three-source models it appears unlikely that gestating adult female gray seals specialize on commercially important fish as all ‘demersal fishes’ account for only 55–65% the predicted diet (Table 4).

For pregnant females, these results indicate a diverse prey assemblage geared towards opportunistic foraging. They also point to a potential bias of previous scat analysis: that they may underrepresent soft-bodied prey such as cephalopods and cartilaginous fishes such as winter and thorny skates [9,10,12,66]. This discrepancy may be accounted for in two ways. First, identification from hard parts can be difficult for both cephalopods and skates. Harvey [20] found that in a captive feeding study of the harbor seal, *Phoca vitulina richardii*, there was only a 37% recovery rate of cephalopod beaks. However, a more recent study of the harbor seal determined a successful recovery rate of 89% for cephalopod beaks [67]. Size of the squid prey may play a role, with large beaks more likely to remain in the stomach or be regurgitated [67]. Cephalopod beaks rarely fully break down but even if they are found in the feces they can be difficult to identify to species [1,13,20]. For skates, denticles are often the only hard part to survive digestion. It can also be difficult to accurately determine species, number of individuals consumed, or prey size from denticles alone [9,68].

Past studies of gray seal diet based on scat analysis have estimated up to half of diet may constitute sand lance [9,12,69]. In contrast, the results of our analysis suggest the contribution of sand lance to the diet of pregnant female gray seals is closer to 10–15% and likely does not exceed one third. This difference may be accounted for both by a more evenly distributed generalist diet and a greater reliance on previously underestimated prey items such as cephalopods and skates. In addition, this discrepancy may occur because scat analysis likely is representative of in-shore foraging near the collection site [9]. Sand lance is primarily an in-shore species in New England, and it therefore is unsurprising that scat analysis shows the greatest reliance on this species [9,12]. Foraging time frames captured by the longitudinal vibrissae and lanugo samples include time spent both offshore and inshore during the summer and fall [35,39,70]. Longfin squid and Shortfin squid are typically found offshore during the spring and come inshore during the summer [71,72]. Thorny skate are typically found offshore in waters 50-100m deep [73]. If throughout the timeline of gestation, gray seals are feeding both inshore and offshore it may have led to the observed lower reliance on highly inshore species such as sand lance in our analysis.

### Conclusions

Determining specific prey sources for this generalist predator remains difficult with stable isotope analysis. Our work has shown that the large number of isotopically similar species gray seals consume limits the ability of SIMMs in predicting robust diet estimates at high taxonomic resolution. Even so, combining prey sources into ecologically informative groups and integrating prior information into SIMMs it is possible to obtain more robust insights into the diet of gray seals though with a loss of taxonomic resolution. Furthermore, while adding *a priori* information from independent estimates of gray seal diets has the potential to further refine SIMMs, they may also induce additional biases into model results. Finally, we were are able to establish the use of vibrissae and lanugo interchangeably as viable proxies for maternal signature and caution that the TEFs for *in-utero* growth for this phocid species likely differ from those determined experimentally in adults. With the gray seal population in New England continuing to grow, it is clear that accurate estimates of their diet will be necessary to accurately predict their impact on both regional fisheries and ecology [74]. As such we recommend that future studies in this region should seek to combine isotopic approaches with other, independent measures of diets such as fatty acid, scat, stomach, and prey DNA analyses to better account for the differing challenges and inherent biases found across methods.
